# Availability and Affordability of Drugs With a Conditional Approval by the European Medicines Agency; Comparison of Korea With Other Countries and the Implications

**DOI:** 10.3389/fphar.2018.00938

**Published:** 2018-09-03

**Authors:** Hye-Young Kwon, Hyungmin Kim, Brian Godman

**Affiliations:** ^1^Division of Biology and Public Health, Mokwon University, Daejeon, South Korea; ^2^Korean National Health Insurance Service, Wonju, South Korea; ^3^College of Pharmacy, Seoul National University, Seoul, South Korea; ^4^Strathclyde Institute of Pharmacy and Biomedical Sciences, University of Strathclyde, Glasgow, United Kingdom; ^5^Division of Clinical Pharmacology, Department of Laboratory Medicine, Karolinska Institutet, Karolinska University Hospital Huddinge, Stockholm, Sweden; ^6^Health Economics Centre, Management School, University of Liverpool, Liverpool, United Kingdom; ^7^Department of Public Health Pharmacy and Management, School of Pharmacy, Sefako Makgatho Health Sciences University, Pretoria, South Africa

**Keywords:** access, affordability, conditional approval, EMA, Korea, reimbursement

## Abstract

**Introduction:** There have been concerns with the availability and affordability of EMA’s recently approved medicines with a conditional approval in Korea. This needs to be addressed to provide future guidance to the authorities in Korea.

**Objective:** Compare the availability and affordability of medicines with a conditional approval by the European Medicines Agency (EMA) among 12 countries (United States, United Kingdom, France, Germany, Switzerland, Italy, Japan, Canada, Taiwan, Australia, New Zealand, and Korea) in light of access to medicine concerns in Korea.

**Methods:** Thirteen medicines were selected and compared in terms of their availability and affordability across 12 countries. Approval rate for the selected medicines and time lag to approval on the basis of EMA’s approval dates were calculated. Reimbursement status and prices were compared as proxies of affordability.

**Results:** The average approval rate was 31.9% for the selected medicines for all countries outside the EU countries. The highest rate was in United States (69.2%) followed by Korea and Switzerland (46.5%). An average of 238 days was taken among the countries for approval. The United States (median: -355 days) was the country where the medicines were most rapidly approved. Korea (152 days) ranked the fifth most rapidly approving country. An average listing or reimbursement rate for all countries was 54.1%. The United States ranked 100% for the listing of their approved medicines followed by Germany (92.3%). Korea (66.7%) ranked eighth. Price dispersion ranged from 1.1 to 3.1. Korean prices of the selected medicines were found to be neither high nor low when compared to other countries.

**Conclusion:** Korea was found to be a country where marketing authorization for more medicines tended to be made and subsequent reimbursement and pricing were not rigid even generous compared to other Asian-pacific countries. Korean drug benefit policies for listing and pricing did not appear to hinder access to medicines even with a conditional approval in comparison with others.

## Introduction

Unlike resource limited countries, access to essential medicines is well-established in high income countries (Hogerzeil, 2004). The concept of essential medicines for high income countries is though still controversial (Hogerzeil, 2004; Duong et al., 2015). However, there have been good examples such as the ‘Wise List’ in Stockholm County Council, Sweden, with high adherence to a limited list of medicines that cover over 90% of the needs of patients in ambulatory care (Gustafsson et al., 2011; Bjorkhem-Bergman et al., 2013; Eriksen et al., 2017). These high adherence rates in Sweden have been achieved through the introduction of robust processes for medicine selection coupled with a comprehensive communication program (Godman et al., 2009; Bjorkhem-Bergman et al., 2013; Eriksen et al., 2017, 2018). Similar examples exist in other countries (Bjorkhem-Bergman et al., 2013). Having said this, providing medicines which meet the priority needs of the community through reliable health systems was a key principal for access to medicines (Hogerzeil, 2004).

Access to medicines can be assessed by their availability and affordability (World Health Organization (WHO), 2010). Availability refers to marketing authorization, available stocks, and distribution (World Health Organization (WHO), 2011a; Erginel, 2014); with affordability closely related to the price of medicines and economic subsidies of healthcare systems. More specifically, copayments for medicines directly affects the use of medicines (Godman et al., 2014; Putrik et al., 2014; Simoens and Sinnaeve, 2014; Kostic et al., 2017), with the degree of coverage for pharmaceuticals depending on the country, the types of medicines and the socioeconomic status of the beneficiaries (Niëns et al., 2012; Niëns and Brouer, 2013).

Korea is a country that achieved universal health coverage in 1989. As the growth of pharmaceutical expenditure in Korea has been greater than other health expenditures (16.3 vs. 15.2% as of 2006), and the portion of drug spending to total health expenditures was greater (21.4% as of 2015) compared to OECD countries (16.3%) (Organisation for Economic Co-operation and Development [OECD], 2012; Ministry of Health and Welfare (MOHW), 2015), policy makers and regulators in Korea have mainly focused on drug cost containment policies in recent years. As part of this process, a positive list system (PLS) was introduced in 2007 to select medicines to be reimbursed based on their cost-effectiveness to the National Health Insurance (NHI). Since the introduction of the PLS, non-reimbursable medicines have been officially allowed to be prescribed with no restrictions but associated with co-payments. In particular, high cost medicines, which hardly met the agreed incremental cost-effectiveness ratio (ICER) threshold were not reimbursed but could still be prescribed for treatment with the costs charged to the patient. As a result, issues of patients’ access to medicines have been raised especially for high cost medicines such as anticancer agents and those for rare diseases where co-payments are an issue (Korean Research-based Pharma Industry Association [KRPIA], 2016). This controversy has influenced Korean drug policy with the government now taking a more liberal attitude towards selecting medicines for reimbursement through measures including a flexible application of ICER thresholds, the exemption of cost-effectiveness appraisals for some medicines, exemptions of price negotiations for some medicines as well as the introduction of risk sharing arrangements (Ministry of Health and Welfare in Korea (MOHW), 2013; Ministry of Health Welfare in Korea (MOHW), 2014). Despite these measures, patients’ advocacy groups and pharmaceutical companies are still appealing to switch back to the negative list system under the name of patient’s rights of access to medicines (Korean Research-based Pharma Industry Association [KRPIA], 2016).

For the interest on public health, conditional approval can be available when the benefit of the immediate availability of medicines outweighs the risk with less comprehensive data than is normally required based on marketing authorization guidelines. The European Medicines Agency (EMA) grants conditional approval for medicines that are aimed at treating, preventing, or diagnosing, seriously debilitating or life-threatening diseases or intended for use in emergency situations or designated as orphan medicines (European Medicines Agency [EMA], 2017, 2018b). Consequently, medicines with a conditional approval were seen as urgently addressing the unmet needs of patients and/or the treatment of severe conditions. These medicines were selected as a subject to address access to medicine issues in Korea given the ongoing controversies that exist in Korea.

In view of this, the purpose of this study is to explore the availability and affordability of EMA’s recently approved medicines with a conditional approval in Korea by comparing the situation in Korea with 11 other countries that are typically referenced by Korea. Subsequently, to review the findings and their implications for the Korean pharmaceutical pricing and reimbursement system in light of their access to medicines. Having said this, there have been concerns with the conditional authorization system in Europe (Banzi et al., 2015). The conditional approval system is different to the recently discussed EMA Adaptive Pathways process intended to also accelerate the uptake of new innovative medicines into routine clinical care in Europe, where there have also been concerns (Ermisch et al., 2016; Vella Bonanno et al., 2017).

## Materials and Methods

### Study Subjects

Considering the severity of diseases and importance of access to new innovative medicines that appear to address unmet need in these critical areas, we collected the list of medicines with conditional approval by EMA from 2014 to 2017 (European Medicines Agency [EMA], 2018a). If these medicines are approved and reimbursed given typically limited clinical data, this would suggest high levels of availability and affordability of new premium priced medicines. Accordingly, a total 14 drugs were selected for this study, and of these all were designated as orphan drugs as well-except for the Pandemic Flu vaccine. We subsequently excluded the Pandemic Flu Vaccine because it is not part of the NHS system in some countries, i.e., the influenza vaccine was provided via the National Immunization Program, which is funded by a general tax in Korea, Japan and Taiwan (Nakatani and Sano, 2002; Lee and Choi, 2008; Tang et al., 2011; Saitoh and Okabe, 2012), and it is currently not reimbursed among a high number of countries involved in this research. This left 13 medicines for analysis.

### Country Comparison

Access to the selected medicines in 12 countries were compared. These countries were the United States, United Kingdom, France, Germany, Switzerland, Italy, Japan, Canada, Taiwan, Australia, New Zealand, and Korea. They were specifically chosen as most of them are officially referenced by the Korean reimbursement and pricing authorities. In addition, all countries except the United States ensure access to medicines through their national healthcare systems.

We searched official websites of each country to collect information on market approval, reimbursement status and the fixed prices of the selected medicines prior to any confidential discount.

### Availability: Approval and Time Lag to Approval

Marketing approval is the first step to launching new medicines across countries. Without market authorization, medicines are not available in a country. For the EU (European Union) member countries, approval of medicines is typically made by the EMA through a centralized process. All other countries have their own authorities for the marketing authorization of medicines such as the Food and Drug Administration (FDA) for United States and the Ministry of Food, Drug and Safety (MFDS) for Korea. We checked the approval rate of the selected medicines for all countries except European countries.

We also checked the moment of licensing in other than EU countries and calculated the time lag to approval on the basis of EMA’s approval date. Although market authorization applications are initiated by manufacturers, time lag to approval represents the delay of the availability of these new medicines across countries.

### Affordability: Reimbursement Status and Prices

Reimbursement status and prices of the selected medicines were considered as proxies of affordability. However, we are aware that the different countries have different processes and systems for the pricing and reimbursement of medicines including whether reimbursed prices are based on the perceived level of innovation versus current standards or economic measures such as ICERs with or without agreed threshold levels (Paris and Belloni, 2013; Godman et al., 2015, 2016; World Health Organization (WHO), 2015). Whether the medicines were listed by national healthcare system was checked from the public information sources (see **Box 1**). For the United States, we checked both the Redbook and the Federal Supply Schedule (FSS).

**Box 1. Data source.**

**Table:** 

Country	Source	Type of prices
United Kingdom	MIMS	Ex-factory pharmacy price (Patented Medicine Prices Review Board in Canada [PMPRB], 2017)
United States	Redbook	Ex-factory pharmacy price (Patented Medicine Prices Review Board in Canada [PMPRB], 2017)
	FSS	Contract price for qualified government (Organisation for Economic Co-operation and Development [OECD], 2008)
France	VIDAL	Ex-factory wholesale price (Patented Medicine Prices Review Board in Canada [PMPRB], 2017)
Italy	www.codifa. it (L’Informatore Farmaceutico)	Ex-factory wholesale price (Patented Medicine Prices Review Board in Canada [PMPRB], 2017) Ex-factory hospital price for hospital drugs (Patented Medicine Prices Review Board in Canada [PMPRB], 2017)
Germany	Rote-Liste	Ex-factory wholesale price (Patented Medicine Prices Review Board in Canada [PMPRB], 2015)
Swiss	Federal Office for Public Health	Ex-factory wholesale price (Patented Medicine Prices Review Board in Canada [PMPRB], 2017)
Canada	Ontario drug benefit formulary	Ex-factory price (Ontrario Ministry of Health and Long-term care in Canada, 2018)
Australia	PBS	Dispensed price for maximum amount (Australian Government Department of Health, 2018)
New Zealand	Pharmac	Ex-factory price (Pharmaceutical Management Agency in New Zealand [PHARMAC], 2018)
Japan	MHLW	Ex-factory pharmacy price (The Federation of Japan Pharmaceutical Wholesalers Association [JPWA], 2014)
Taiwan	NHIA	Public Price
Korea	HIRA	Ex-factory pharmacy price


*MIMS, Monthly Index of Medical Specialties; FSS, Federal supply schedule; PBS, Pharmaceutical Benefits Scheme; Pharmac, Pharmaceutical management agency; MHLW, Ministry of Health, Labour and Welfare; NHIA, National Health Insurance Administration; HIRA, Health Insurance Review & Assessment Service.*

For price comparisons, we used public prices for reimbursed medicines. Public prices do not generally reflect effective prices since substantial rebates or discounts are being offered to insurers in many countries to enhance reimbursement for new premium priced medicines. These confidential arrangements between manufacturers and payers are increasingly practiced (Vogler et al., 2012; Ferrario and Kanavos, 2013, 2015; Ferrario et al., 2017; Pauwels et al., 2017). However, published prices are the only publicly available information for price comparisons. We compared prices of the selected medicines with this limitation and careful interpretation. Each country publishes prices online and types of prices are different, for examples, ex-factory prices, ex-factory wholesale price and ex-factory pharmacy price. Types of price and the source of price information for each country are shown in **Box 1**.

## Results

**Table 1** shows the brand-name with active pharmaceutical ingredients and the main indications of the 13 selected medicines approved by EMA during 2014–2017. As of March 16, 2018, the average number of countries in which the selected medicines were licensed was three out of eight (except four European countries). Blinatumomab (Blincyto^TM^) and Ixazomib (Ninlaro^TM^) had been approved in six countries while Holoclar^TM^, Translarna^TM^ (Ataluren), and Zalmoxis^TM^ were not yet approved. Blinatumomab was listed in eight countries while Zalmoxis^TM^ was not yet reimbursed.

**Table 1:** Basic information of the selected medicines.

Brandname	API	Main indication	Approval date(EMA)	No. of authorized countries^a^	No. of reimbursed countries^b^
Bavencio^TM^	Avelumab	MCC	18 September 2017	9 (5)	5 (41.7%)
Blincyto^TM^	Blinatumomab	ALL	23 November 2015	10 (6)	8 (66.7%)
Cometriq^TM^	Cabozantinib	Medullary thyroid carcinoma	21 March 2014	8 (4)	6 (50.0%)
Deltyba^TM^	Delamanid	MDR-TB	23 April 2014	6 (2)	6 (50.0%)
Holoclar^TM^	human corneal cells with stem cells	limbal stem cell deficiency	17 February 2015	4 (0)	2 (16.7%)
Lartruvo^TM^	Olaratumab	soft tissue sarcoma	9 November 2016	8 (4)	6 (50.0%)
Natpar^TM^	Parathyroid hormone	chronic hypoparathyroidism	24 April 2017	5 (1)	3 (25.0%)
Ninlaro^TM^	Ixazomib	multiple myeloma	21 November 2016	10 (6)	6 (50.0%)
Ocaliva^TM^	Obeticholic acid	primary biliary cholangitis	12 December 2016	5 (1)	5 (41.7%)
Sirturo^TM^	Bedaquiline	MDR-TB	5 March 2014	9 (5)	6 (50.0%)
Translarna^TM^	Ataluren	Duchenne muscular dystrophy	31 July 2014	4 (0)	4 (33.3%)
Venclyxto^TM^	Venetoclax	CLL	5 December 2016	9 (5)	5 (41.7%)
Zalmoxis^TM^	Allogeneic T cells	HSCT	18 Auguest 2016	4 (0)	0 (0.0%)


*MDR-TB, multidrug resistant tuberculosis; ALL, acute lymphoblastic leukemia; HSCT, haploidentical haematopoietic stem cell transplantation; CLL, chronic lymphocytic leukaemia; MCC, metastatic Merkel cell carcinoma. ^*a*^Maximum number of countries is 12 and 8 between brackets, which excluded EU countries. ^*b*^Number of countries where the selected medicines were reimbursed, % out of 12 in brackets.*

### Approvals by County

**FIGURE 1:**
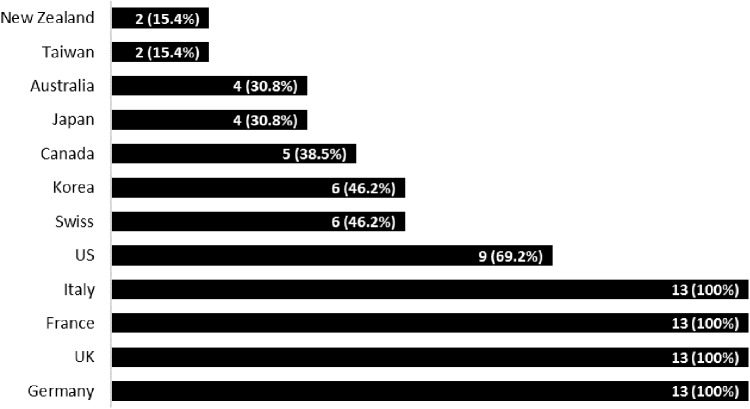
Number of approved medicines and approval rates by country. NB: Brackets = % of approved medicines out of 13.

On average, there was a 31.9% approval rate for the 13 medicines for all countries except the EU countries. The approval status for each country is depicted in **Figure 1**. As EMA approval was a reference, all the included EU countries had approval for the 13 medicines while Switzerland had approved only six medicines. The highest rate of approval was shown in United States (69.2%, 9 out of 13) followed by Korea (46.2%) and Switzerland (46.2%, 6 out of 13 products). Taiwan and New Zealand showed the lowest approval rate of 15.4% (2 out of 13 products). Among Asia pacific countries, Korea was a country where more medicines were available than other countries.

### Time Lag to Approval

**FIGURE 2:**
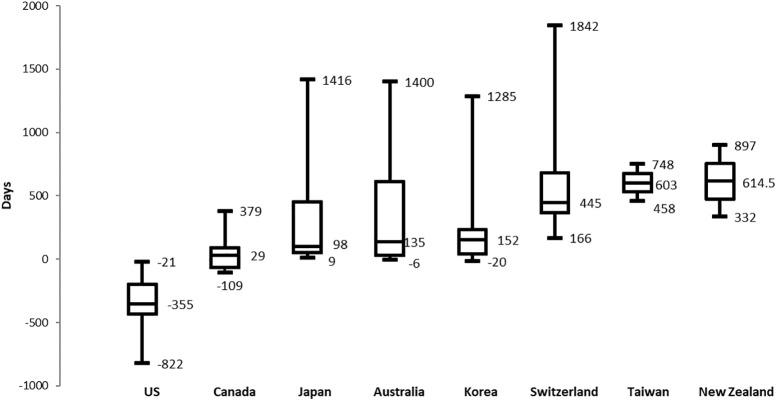
Box-plot of time lag to approval on the basis of EMA (Maximum, Median, Minimum values).

As shown in **Figure 2**, the time lag to approval was calculated on the basis of EMA’s approval date and compared among the eight other target countries. An average of 238 days (median: +120 days) were taken among the countries for approval. The United States (median: -355 days) was the country where all medicines were most rapidly approved followed by Canada (median: +29 days) and Japan (median: +98 days).

Korea (median: +152 days) was ranked the fifth most rapidly approving country. Unlike EU countries, Switzerland had taken a relatively long time for approval. Taiwan and New Zealand showed 603 and 614.5 days respectively for the time lag from the EMA. Korea, Japan, and Autralia had the greatest variations between maximum and minimum values while the United States received FDA’s approval before the EMA’s approval date. Unlike the approval rate, Korea took a relatively long time to approve the selected medicines compared to Japan and Australia.

### Reimbursement Status

**FIGURE 3:**
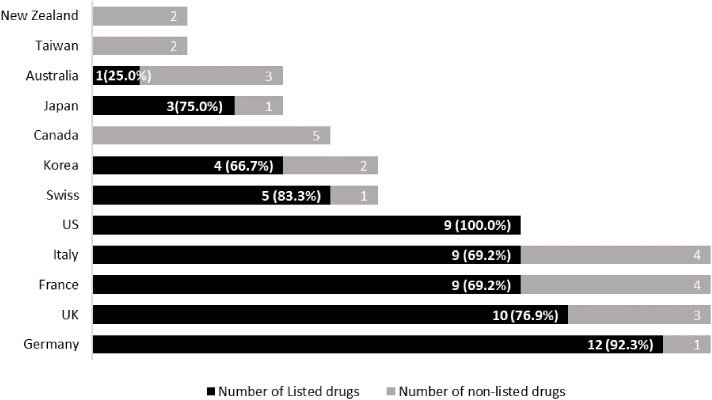
Number of reimbursed medicines and listing rate by country. NB: Redbook was referenced for the United States and brackets = % of reimbursed (listed) medicines out of those approved.

Among the approved medicines, an average reimbursement rate for the 12 countries was 54.4%. Although all medicines were approved in European countries, listing (reimbursement) rates varied greatly across the countries as shown in **Figure 3**. Germany (92.3% of the 13 medicines) showed a relatively high rate of reimbursement followed by the United Kingdom (76.9%) whereas Switzerland, whilst in Europe but not part of the EU, only reimbursed five out of six approved medicines (83.3%).

Overall, the United States ranked 100% of listing of the nine approved medicines based on the Redbook. However, eight out of nine medicines were listed in the FSS (88.9%). Germany (92.3%, 12 out of 13) ranked 2^nd^ followed by Switzerland (83.3%, five out of six), United Kingdom (76.9%, 10 out of 13), Japan (75.0%, three out of four), as well as France and Italy (69.2%, 9 out of 13). Korea ranked eighth with a listing rate of 66.7% (of 6) followed by Australia (25.0% of 4). Canada, Taiwan, and New Zealand listed none of the medicines. Within Asia-Pacific region, Japan showed the highest rate of reimbursement followed by Korea.

### Price Comparisons

**FIGURE 4:**
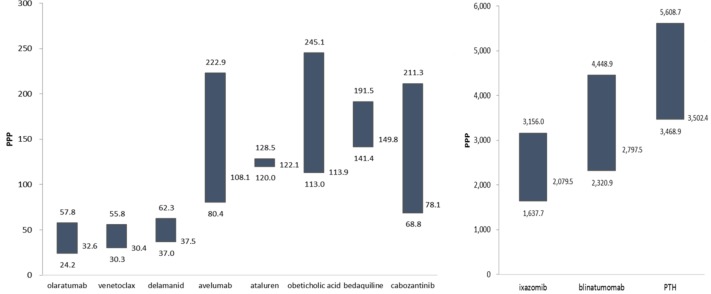
Prices of the medicines with a conditional approval (PPP_USD, Maximum, Median, Minimum values). Holoclar was not depicted as German price was unidentified. Its price of 120,396 (PPP) in Italy was not depicted.

**Figure 4** depicts the prices of each medicine for all the countries. Prices were adjusted with Purchasing Power Parity (PPP_USD). Although the types of prices differed by country and direct comparison was controversial, it was found that price dispersion was broad with some medicines such as Cabozantinib, Avelumab, Olaratumab and Obeticholic acid. However, there was limited price dispersion with Ataluren. The price dispersion, calculated by the maximum price divided by the minimum price, ranged from 1.1 to 3.1. Even in the same country such as the United States, the prices from the Redbook and FSS differed. On average, the Redbook prices (AWPs) were 1.7 times [1.1∼1.8] higher than the FSS prices. Price dispersion and the number of listed countries were significantly correlated (ρ = 0.704, *p* = 0.0072). Thus, the more countries the drug is listed, the greater the difference in drug prices. This proves that the one price policy of drug companies is not typically applied empirically.

**Table 2** shows the prices of four medicines listed in Korea compared to the other countries. Although direct comparisons of prices with different attributes should be done carefully, the price level of Korea was estimated with this limitation. The prices of Blinatumomab ranged from 0.8 to 1.6 times of the Korean price and those of Olaratumab ranged 1.3 to 2.4 times. For bedaquiline (0.8∼1.1 times) and delamanid (0.6∼1.4 times), Korean prices were found to be relatively high in consideration of compared countries’ GDP level.

**Table 2:** International price comparison of Korea’s reimbursement drug (USD PPP).

Countries		Bedaquiline	Delamanid	Blinatumomab	Olaratumab	Reference
US	Redbook	191 (1.1)	–	4,449 (1.6)	57.8 (2.4)	Ex-factory pharmacy price
	FSS	–	–	2,385 (0.8)	36.0 (1.5)	Contact price for government
UK		141 (0.8)	37 (0.8)	2,867 (1.0)	54.0 (2.2)	Ex-factory pharmacy price
France		–	37 (0.8)	3,293 (1.2)	–	Ex-factory wholesale price
Italy		150 (0.8)	39 (0.9)	3,582 (1.3)	34.9 (1.4)	Ex-factory wholesale price
Swiss		–	–	2,321 (0.8)	–	Ex-factory wholesale price
Germany		180 (1.0)	38 (0.8)	2,864 (1.0)	32.6 (1.3)	Ex-factory wholesale price
Japan		–	62 (1.4)	–	–	Ex-factory pharmacy price
Australia		–	–	2,724 (1.0)	–	Dispensed price for max amount
Korea		180 (1)	45 (1)	2,822 (1)	24.2 (1)	Ex-factory pharmacy price


## Discussion

Access to medicines has been a common issue for among low and middle-income countries (Cameron et al., 2009). Even high income countries are now struggling to fund new premium priced medicines whilst maintaining universal access, which has resulted in new models to improve their managed entry (Godman et al., 2012, 2015, 2016; Malmstrom et al., 2013; Paris and Belloni, 2013).

This study explored access to medicines, particularly issues surrounding the Korean context in terms of the availability and affordability of recently approved medicines in areas of high unmet need. Thirteen medicines with conditional approval in the EU were selected for their urgency and necessity to address patients’ unmet needs, with a 37.5% approval rate among eight countries excluding the four European countries. An average time lag to approval was 238 days from EMA’s approval date. The United States with a 69.2% approval rate was always a country that manufacturers attempted to obtain approval before EMA approval. Among the 13 EMA approved medicines, an average reimbursement rate for the 12 countries was 54.4%. Some countries including Australia, Canada, Taiwan, and New Zealand, tended to be laggards to approve and reimburse the selected medicines.

The range of prices was found to be broad among most of the researched medicines. This is contrary to a one price policy as a global pricing strategy (World Health Organization (WHO), 2011b; Young et al., 2017), which has been often claimed by global pharmaceutical companies. However, some publications have shown considerable variation in the prices of patented medicines across countries (Leopold et al., 2013; Vogler et al., 2015), similar to our findings, with the price of medicines varying depending on issues such as national reimbursement policies and economic levels (Garau et al., 2011; Paris and Belloni, 2013; Towse et al., 2015; Godman et al., 2016). In addition, as mentioned, we are seeing the rise in managed entry agreements across countries to increase access to new medicines, which often contain confidential discounts (Vogler et al., 2012; Ferrario and Kanavos, 2013, 2015; Godman et al., 2016; Ferrario et al., 2017; Pauwels et al., 2017) making price comparisons difficult.

Encouragingly, Korea ranked second in the approval rate of 46.5% excluding EU countries and fifth in the time lag of 152 days [-20∼1,285]. The reimbursement rate was 66.7% of the approved medicines, with the prices of the selected medicines neither high nor low in comparison with the other countries.

We are aware this study has limitations. Firstly, we selected medicines with a conditional approval. These medicines were considered exceptional compared to others, which normally go through standard procedures of marketing approval and reimbursement appraisal. As we explored the states of approval and reimbursement of these medicines across the board, it may be difficult to generalize the results to other medicines. Secondly, published price information differed across the countries and it can be problematic to directly compare them. However, we could not adjust prices for comparison especially as some countries adopt risk sharing schemes or clawback systems for new medicines and published prices do not reflect these adjusted prices (Ferrario and Kanavos, 2013; Ferrario et al., 2017). In view of this, we had to approximately compare Korean prices with others to find some implications. In addition, we did not consider other funds besides the pharmaceutical benefit schemes, e.g., Blinatumomab was not covered by Ontario Drug Benefit formulary although it did through NDFP (New Drug Fund Program) (Cancer Care Ontario in Canada (CCO), 2017).

Nevertheless, this study demonstrated contradictory results to pharmaceutical company’s one pricing strategy (World Health Organization (WHO), 2011b) since most of the studied medicines showed a wide range of price distribution except Ataluren. Our study though reconfirmed the strategy that pharmaceutical companies try to delay the launch of their new medicine in countries with traditionally low prices or even not market at all in countries that are referenced by other countries with larger markets (Leopold et al., 2013; Vogler et al., 2015; Young et al., 2017), exacerbated particularly in Europe by extensive reference pricing across countries (Leopold et al., 2012). The United States showed 100% reimbursement of approved medicines and the highest prices while Taiwan and New Zealand were delayed in both their approval and reimbursement of the selected medicines.

As a result, the availability and affordability of the selected medicines featured geographically different aspects. Asia-pacific countries that are mostly small markets, or try to attain lower prices, were delayed in their approval and reimbursement rates for these selected medicines compared to other continents. However, Korea performed better than other selected Asian-Pacific countries for these medicine, which is encouraging given ongoing concerns.

## Conclusion

Korea was found to be a country where marketing authorization for more these selected medicines with EMA conditional reimbursement tended to be made, and subsequent reimbursement and pricing were not rigid even generous compared to other Asian-pacific countries. As a result, contrasting with the arguments of patient’s advocacy groups and pharmaceutical companies in Korea (Korean Research-based Pharma Industry Association [KRPIA], 2016). Consequently, Korean drug benefit policies for listing and pricing did not appear to hinder access to medicines even with a conditional approval in comparison with other countries. This is to be encouraged.

## Author Contributions

HK and H-YK developed the concept of the paper and undertook the analysis. All the authors were involved with developing and refining the manuscript and approved the final draft before submission.

## Conflict of Interest Statement

HK is employed by the Korean National Health Insurance Service. The remaining authors declare that the research was conducted in the absence of any commercial or financial relationships that could be construed as a potential conflict of interest.
